# Does a biological invasion modify host immune responses to parasite infection?

**DOI:** 10.1098/rsos.240669

**Published:** 2025-01-15

**Authors:** Gregory P. Brown, Richard Shine, Lee A. Rollins

**Affiliations:** ^1^ School of Natural Sciences, Macquarie University, Sydney, NSW 2109, Australia; ^2^ Evolution & Ecology Research Centre, School of Biological, Earth and Environmental Sciences, University of New South Wales, Sydney, NSW 2052, Australia

**Keywords:** *Bufo marinus*, local adaptation, ecoimmunology, leucocytes, phagocytosis, bacteria-killing

## Abstract

Biological invasions can disrupt the close and longstanding coevolved relationships between host and parasites. At the same time, the shifting selective forces acting on demography during invasion can result in rapid evolution of traits in both host and parasite. Hosts at the invasion front may reduce investment into costly immune defences and redistribute those resources to other fitness-enhancing traits. Parasites at the invasion front may have reduced pathogenicity because traits that negatively impact host dispersal are left behind in the expanding range. The host’s immune system is its primary arsenal in the coevolutionary ‘arms race’ with parasites. To assess the effects of invasion history on immune responses to parasite infection, we conducted a cross-infection experiment which paired common-garden reared cane toads and lungworm parasites originating from various sites in their invaded range across northern Australia. Infected toads had larger spleens and higher concentrations of eosinophils than did uninfected toads. Infected toads also exhibited lower bacteria-killing ability, perhaps reflecting a trade-off of resources towards defences that are more specifically anthelminthic. The impact of infection intensity on multiple immune measures differed among toads and parasites from different parts of the invasion trajectory, supporting the hypothesis that invasion has disrupted patterns of local adaptation.

## 1. Introduction

Biological invasions can produce rapid and dramatic divergence of traits at the expanding edge of the range [1]. The occurrence of genetic bottlenecks and founder effects, for instance, can reduce genetic variance and limit trait frequencies at the invasion front [2,3]. In addition, many suites of traits that enhance dispersal become elaborated at the invasion front, often at the expense of other traits (e.g. reproduction) through life history or energetic trade-offs [4–6]. Traits related to immune surveillance and response can also be affected by invasion. If native range pathogens and parasites are left behind, immune investment may be decreased or economized, and invasive populations may shift away from costly specific immune reactions towards more general and economical ones [7]. But if novel parasites and pathogens are encountered, immune surveillance and appropriate responses may instead become elevated at the invasion front (the expanding edge of the invasive range) compared with longer established populations at the core of the range [8,9].

Parasites are under strong selection to successfully locate and infect their host, and hosts are concurrently under selection to prevent infections or reduce their negative effects. Thus, the conflict between host and parasite is a classic example of antagonistic coevolution (i.e. an evolutionary arms race [10]). Spatial structure in host and parasite populations introduces added complexity to this coevolution. Relative rates of gene flow in both host and parasite populations can influence the local outcomes of their interactions. Higher levels of gene flow should act to increase genetic diversity for selection to act upon [11]. As a result, local adaptation may be apparent either in the parasite (better able to infect sympatric than allopatric hosts) or in the host (better able to resist sympatric parasites better than allopatric ones) [10–14].

Still further complexity in host–parasite interactions arises if the spatial structuring of their populations is disrupted, such as through species translocation or a continued period of range expansion [12,13,15,16]. Two major changes relevant to host–parasite interactions are likely to occur during biological invasions. First, both natural selection and spatial sorting may favour reduced investment in costly immune defences by hosts at the invasion front [7,17,18]. At the forefront of the invasion, for example, low host densities limit transmission opportunities [1,19]. Relaxed selection on anti-parasite defences would allow reallocation of resources away from immunity (and hence reduce host resistance to infection), but towards other traits (e.g. growth, reproduction) that would enhance fitness [3,15,20] or enable more rapid dispersal [1].

Second, parasites are likely to exhibit lower virulence and higher infectivity at their host’s invasion front [1,15,21]. By reducing opportunities for transmission, low host density at the invasion front can increase selection on the parasite’s ability to successfully locate and infect a host [22]. Spatial sorting of parasite impacts that impede host dispersal also are likely to result in low pathogenicity at the invasion front [1,3,23,24]. Parasites could reduce host dispersal in several ways [16]; such as by inducing ‘sickness behaviours’ that reduce host energy intake or activity [25,26] or by causing hosts to divert energy into costly immune defences rather than into traits that enhance dispersal.

Under these scenarios, invasion is predicted to produce spatial differences in the type or level of immune response produced by hosts from the front compared with those from core populations in the invasive range [18] as well as spatial differences in the immune responses elicited by parasites from frontal and core populations. We tested these predictions using an iconic invasive species, the Australian cane toad *Rhinella marina* (Linnaeus, 1758) and its coevolved nematode lungworm parasite *Rhabdias pseudosphaerocephala* (Kuzmin, Tkach & Brooks, 2007). Previous studies have also shown that several toad traits including immune measures have shifted, sometimes in a nonlinear manner, as the invasion has progressed across Australia [17,20,27,28]. Lungworm traits have shifted also, as the parasites have been carried westwards across the continent by toads [22,29].

We carried out an experimental cross-infection study, exposing common-garden reared toads whose parents had been collected from different sites across their invasive range to lungworms similarly sourced from different sites across the range. Four months later, we determined the level of parasite infection in each toad and measured a suite of immune-related parameters. Our aims were twofold. First, we wished to compare immune measures between infected and uninfected toads to identify which traits were most affected by the presence of parasites. Second, focusing only on infected individuals, we wished to determine if levels of immune response depended on the respective geographic origins of the host and parasite populations. In Australia, infection success of *R. pseudosphaerocephala* in cane toads depends on the invasion history of both the host and the parasite [22]. Parasites from the invasion front are highly infective to all hosts, regardless of where the toad is from. In long-established populations behind the invasion front, host are better at preventing infections by local sympatric parasites than by more allopatric parasites [22].

Here, we investigate immune-associated measures potentially related to spatial variation in host–parasite interactions, and to determine whether or not the host’s immune response to the final stage (i.e. lung-dwelling) of *R. pseudosphaerocephala* infection was affected by host and parasite origins. Specifically, we predicted that: (i) toads from the invasion front would produce weaker immune responses (a proxy index of lower virulence) to lungworm infection than would toads from range-core populations; and (ii) lungworms from the invasion front would elicit weaker immune responses from their hosts than would lungworms from range-core populations.

## 2. Methods

### 2.1. Study species

**Figure 1.**
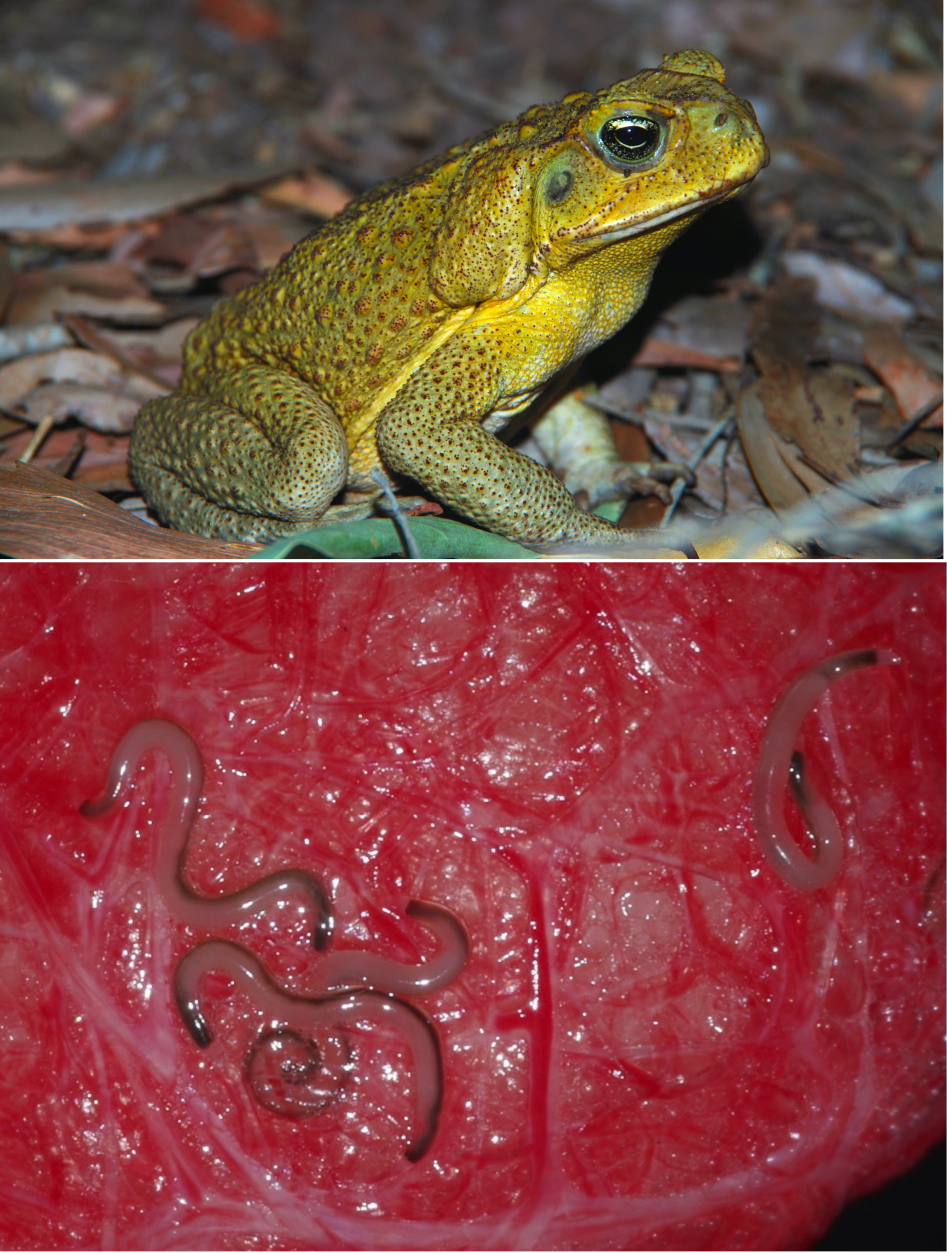
Cane toads (*Rhinella marina*) in Australia (top) are often parasitized by lungworms (*Rhabdias pseudosphaerocephala*) (bottom).

Cane toads (figure 1) were introduced into Australia as a biocontrol strategy to reduce insect pests from sugarcane crops [30]. Among the 101 toads transported from Hawaii, at least one individual contained at least one parasitic nematode in its lungs. *Rhabdias pseudosphaerocephala* (figure 1) parasitize cane toads in their native range in South America [31]. This is the only *Rhabdias* species that infects cane toads in Australia. Although native Australian frogs can be infected with a native lungworm (*Rhabdias hylae*), cross-infections among the Australian and introduced species do not occur [32]. Adult *Rhabdias* have a lifespan of up to five months [33], feeding on blood in the lungs of their host and shedding eggs. *Rhabdias* eggs shed in the lungs are swallowed into the digestive tract and hatch in faeces deposited in the environment. Over the next 5 days, larvae develop into a nonfeeding infective stage (L3) which can survive in the environment for up to two months [34]. If a toad encounters contaminated soil the larvae penetrate through the skin and migrate through tissues to reach the lungs where they mature into adults and the life cycle continues.

### 2.2. Toad collection

**Figure 2.**
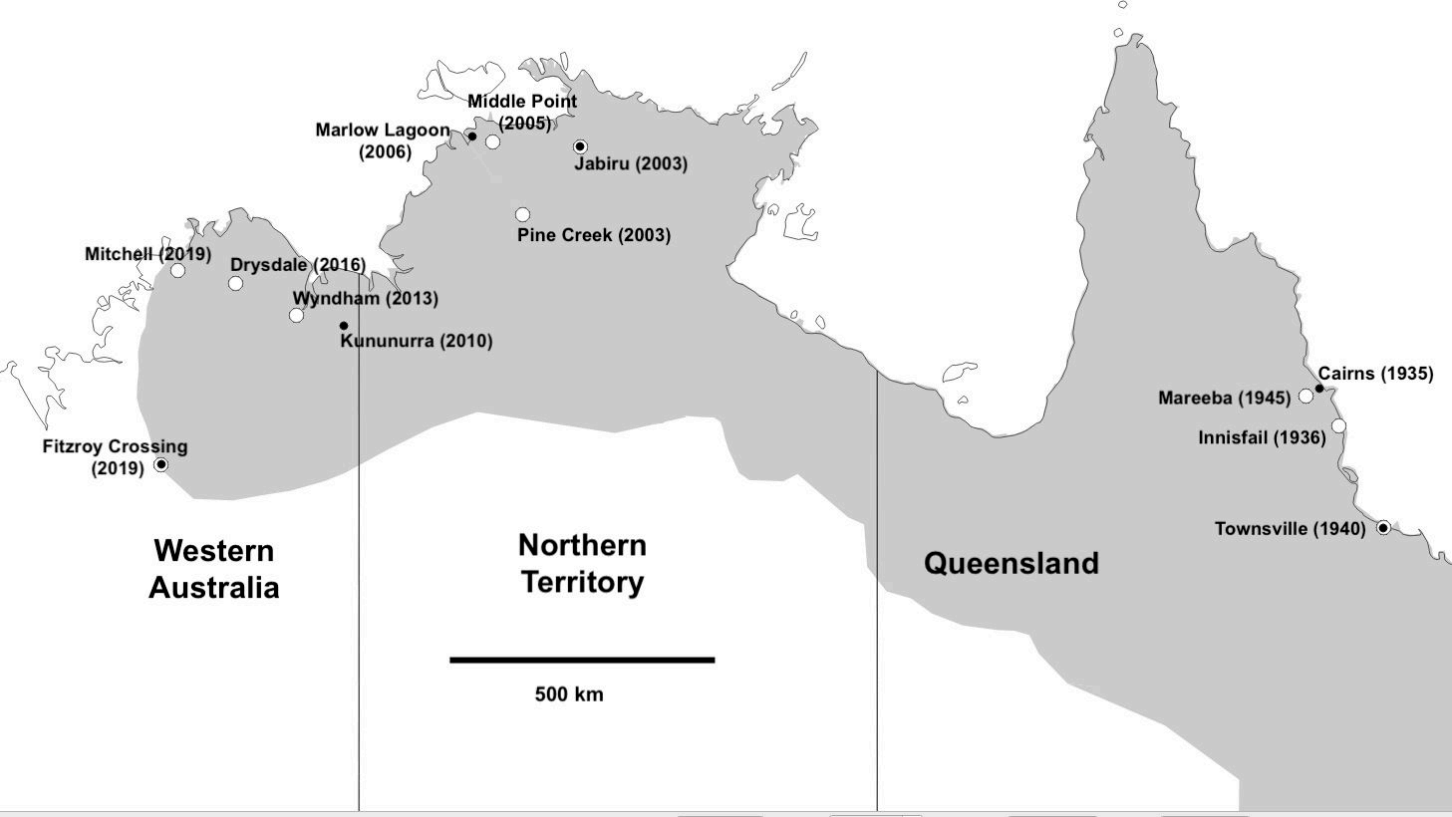
Map showing northern Australian collection sites for parental toads that produced common-garden clutches (white symbols) and lungworms (black symbols). Dates in brackets indicate the year of toad arrival at each site. The grey-shaded area represents the approximate distribution of cane toads in 2020.

In late 2020 we collected 10 male and 10 female adult toads from each of 10 sites across northern Australia (figure 2) to produce common-garden offspring. Three sites were in Queensland (Qld), three sites were in the Northern Territory (NT) and four sites were in Western Australia (WA). At the time of collection, toads had been present at the Qld sites from 75 to 85 years, at the NT sites from 14 to 17 years and at the WA sites for 1 to 10 years. (figure 2). We transported the toads to our field station at Middle Point NT and housed them indoors, grouped by site, in 700 l enclosures.

### 2.3. Breeding and tadpole rearing

Between November 2020 and February 2021, pairs of toads from each site were induced to breed with an injection of leuprorelin acetate (Lucrin; Abbott Australasia, Sydney, Australia). At the time of breeding, none of the parental toads were shedding *R. pseudosphaerocephala* eggs in their faeces. However, they may have been exposed to or infected with *R. pseudosphaerocephala* prior to being collected from the wild. Two clutches were obtained from toads from Mareeba and from Townsville and single clutches obtained from the other eight sites, resulting in a total of 12 clutches. Approximately 100 tadpoles were randomly selected from each clutch and reared in separate 70 l tubs equipped with aerators. Tadpoles were fed daily on frozen lettuce and fish flakes. Half of the water in each tub was changed every other day.

### 2.4. Metamorph rearing

Metamorphs began emerging from tadpole tubs after three weeks. One hundred metamorphs from each clutch were reared in 700 l enclosures lined with dried sand and containing a shallow 40 cm diameter water dish. Toadlets were fed daily on termites mixed with crushed cat food pellets. When they were approximately six months old, they were experimentally exposed to *R. pseudosphaerocephala*.

### 2.5. Lungworm collection

Ten toads infected with *R. pseudosphaerocephala* were collected from each of six sites: two in Qld, two in the NT and two in WA (figure 2). In each state, one of the parasite collection sites was also a site where parental toads used for common-garden breeding were collected. The other sites in each state where parasites were collected were not used as parental toad sites.

The infected toads were brought to the Middle Point field station in the NT (figure 2). Toads were held separately by site in 700 l bins lined with wood chips and containing shallow 40 cm diameter water dish. Toads were fed daily with meal worms mixed with cat food pellets. To obtain infective larvae, fresh faeces were collected from each bin and placed on small square damp paper towel in the middle of a petri dish. Water was added to the paper towel each day to maintain moisture. After 5 days, the petri dish was flooded with clean water and the mobile larvae that emerged from the paper towel were pipetted out and counted.

### 2.6. Experimental infections

We experimentally exposed 228 toads from 12 families to infective *R. pseudosphaerocephala* larvae. Experimental infections were performed by placing each juvenile toad in a 250 ml plastic container lined with damp paper towel. Fifty infective larvae were counted out into 1 ml of water and added to the damp paper towel of each chamber. After 12 h, toads were held above their containers and rinsed with water to remove any larvae remaining on the skin in order to standardize the duration of exposure to larvae. A further 80 toads from the 12 families were used as uninfected controls. These control toads were also held for 12 h in 250 ml cups, but with 1 ml of water that did not contain any *R. pseudosphaerocephala* larvae. After being removed from the plastic cups, toads were measured for snout-to-vent length (SVL) and body mass and moved to larger 15 l individual cages with paper towel flooring and a 200 ml water dish for long-term housing. Cages were cleaned and water and flooring were replaced each time a toad defaecated. This hygiene regime was enacted to prevent self-reinfection from occurring. *Rhabdias pseudosphaerocephala* larvae require 5−7 days of development in faeces in order to become infective. Thus, we immediately removed faeces from cages to prevent any exposure to infective larvae after the initial experimental encounters.

### 2.7. Euthanasia and immune measures

After 108−136 days, toads were euthanized with an overdose of pentabarbitone sodium (Lethabarb, Virbac, Milperra, Australia). Once toads were anaesthetized, we collected 0.3 ml of blood via cardiocentesis into a heparinized sterile syringe. The blood sample was used to provide haematological and immune measures (see below). When toads were dead, we dissected out the lungs and counted any adult lungworms inside of them. Due to time and space constraints, euthanasia and immune measures were carried out over 36 days, with 8–16 toads killed per day.

#### 2.7.1. White blood cell differential count

White blood cells (WBCs) are the effector cells of the immune system; different types play different roles in surveillance and defence. Relative proportions of the different WBC types provide an index of immune configuration. To obtain differential counts of five different WBC types, we prepared a single slide with a thin smear using 5 μl of whole blood. The smear was air dried, mixed in methanol and stained using modified Giemsa solution. A cover slip was placed on top, and the slide examined under a compound microscope at 100×. We scanned each slide systematically, making several longitudinal scans to locate leukocytes and identified them as basophil, eosinophil, lymphocyte, monocyte or neutrophil. We identified 100 leukocytes on each slide to produce differential counts.

#### 2.7.2. Total white blood cell concentration

A 1 : 200 blood dilution was prepared by mixing 5 μl of whole blood with 995 μl of Natt–Herrick solution. This was vortex-mixed and refrigerated overnight. The next morning the solution was vortex-mixed again and 10 μl withdrawn and placed into a Neubauer haemocytometer. The haemocytometer was examined under a dissecting microscope and the numbers of WBCs in four large corner squares of the chamber were counted and the concentration of cells per millilitre whole blood calculated accordingly [35].

We used these total WBC concentration values together with the differential counts of each cell type (see above) to calculate concentrations of basophils, eosinophils, lymphocytes and neutrophils. Monocytes were too rare (absent in 86% of toads) to merit calculation of their concentration or to use as a variable in further analyses.

#### 2.7.3. Phagocytosis assay

We used the assay described by Marnila *et al*. [36] to measure the ability of toad blood to phagocytize foreign particles (zymosan—a glucan from yeast cell walls). We added 37 μl of blood to 703 μl sterile Amphibian Ringers then for each sample added 240 μl of this 1 : 20 blood dilution to three wells of a 96-well plate, along with 30 μl of a 10 mM solution of luminol (Sigma A8511). We added 10 μl of a 10 mg solution of zymosan (Sigma Z4250) in Amphibian Ringers to two of these wells. The third well for each sample served as a negative control so 10 μl of Amphibian Ringers was added to it in place of zymosan solution. The plate was then placed into a luminometer and light emissions were recorded at 4 min intervals for 160 min. We calculated the average luminescence over the 40 observations as an estimate of phagocytosis activity.

#### 2.7.4. Bacteria-killing ability of plasma

Plasma contains soluble proteins (e.g. complement, natural antibody, lysozyme) and we measured its ability to kill *Escherichia coli* (ATCC 8739) using a modification of the method of Matson *et al.* [37]. After samples requiring whole blood were removed from the vial, we centrifuged the remaining blood for 4 min at 14*g* to obtain plasma. We added 23 μl of plasma to 207 μl of CO_2_-independent media enriched with l-glutamine in a sterile 1.5 ml microcentrifuge tube. We dissolved a lyophilized pellet of *E. coli* in sterile phosphate-buffered saline to yield a concentration of approximately 75 colony forming units per microlitre. We added 10 μl of this bacterial suspension to each diluted sample of toad plasma, briefly vortex-mixed it and then spread 50 μl of the bacteria–plasma mixture onto a sterile tryptic soy agar plate as the 0 min sample. The remaining bacteria–plasma mixture was incubated at 25°C for 60 min. After 60 min, a further 50 μl sample was spread onto another agar plate.

To quantify bacterial growth in the absence of toad plasma, we also prepared control samples where 10 μl of *E. coli* suspension was added to 240 μl of CO_2_-independent media that did not contain any toad plasma. We plated out 50 μl of these control samples at 0 and 60 min as well. All agar plates were then incubated at 37°C for 24 h after which the number of *E. coli* colonies growing on each plate was counted.

We used the index of bacteria-killing ability (BKA) formulated by Allen *et al.* [38] to measure the ability of a toad plasma sample to neutralize *E. coli* after 60 min exposure:


BKA=−1×(%changeinCFUplasmasample)–(%changeinCFUincontrolsamples).

This index corrects for changes in bacteria counts observed in control plates and yields positive values for plasma samples that greatly reduce bacteria counts and negative values when little bacteria killing occurs.

### 2.8. Analyses

The study involved 308 toads (80 control, 228 exposed to *R. pseudosphaerocephala*), but sample sizes vary slightly among analyses because some blood samples were too small to complete all the immune measures.

#### 2.8.1. Comparisons between infected and uninfected toads

Our first set of analyses assessed differences in immune measures between 101 toads that had *R. pseudosphaerocephala* in their lungs when they were euthanized and 207 that did not. The 207 uninfected individuals included 80 control toads that had never been exposed to *R. pseudosphaerocephala* larvae as well as 127 toads that were exposed to *R. pseudosphaerocephala* larvae but were parasite-free at the end of the experiment. Preliminary analyses indicated that these two groups of uninfected toads did not differ significantly in any of our immune measures (all *F* < 3.32, all *p* > 0.071; electronic supplementary material, supplementary table 1).

**Table 1.** Mixed model analyses of the effects of *R. pseudosphaerocephala* infection status and host origin on circulating WBC concentrations. Italic font indicates a significance level of *p* < 0.05.

		basophils		eosinophils		lymphocytes		neutrophils	
effect	d.f.	*F*_252_	*p*	*F*_252_	*p*	*F*_252_	*p*	*F*_252_	*p*
SVL	1, 252	5.63	*0.0184*	0.45	0.5038	0.10	0.7518	1.09	0.2973
infected	1, 252	1.24	0.2663	5.77	*0.0170*	0.76	0.3834	0.02	0.8768
host origin	2, 252	2.65	0.0726	7.73	*0.0006*	1.54	0.2164	0.13	0.8809
infected × host origin	2, 252	2.45	0.0879	0.58	0.5606	0.62	0.5377	0.66	0.5152

We used generalized linear mixed models to compare immune measures among these three infection groups. Toad SVL at the end of the experiment was included as a covariate, and infection status and toad state of origin (and the interaction between them) were included as independent variables. We did not include parasite origin as an independent variable in these models because it was undefined for control and uninfected toads. Clutch and toad site (within state) were included as random effects in each model to incorporate potential familial and small-scale geographic similarities in measures. Assay date was also included as a random effect in each model to accommodate any potential for immune measures made on the same day to be similar, either through vagaries of reagent preparation or small differences in infection duration.

#### 2.8.2. Relationships among infected toads only

Our second set of analyses focused only on 101 toads that had become infected following experimental exposure to *R. pseudosphaerocephala* larvae. We analysed the same set of immune-related dependent variables as above but used an expanded set of independent variables. Along with toad state of origin, we included *R. pseudosphaerocephala* state of origin and *R. pseudosphaerocephala* infection intensity (number of adult worms in the lungs at dissection) and all interactions among these three variables. Toad body size (SVL) was included as a covariate in all models.

Again, we used clutch ID, assay date and toad site (within state) as random effects to model familial, temporal and fine-scale geographic similarities among toads. In these analyses limited to infected toads, we also included worm site (within state) as a random effect to incorporate fine-scale geographic similarities in parasite effects. We also included assay date as random effect, as above. All analyses were run using Proc Glimmix in SAS 9.4 (SAS Institute, Cary, NC). We used a normal distribution with identity link function for spleen size and BKA. For analyses of all other dependent variables, we used a lognormal distribution and identity link function. To validate that our models met assumptions of generalized linear analysis, we checked the residuals for normality and homogeneity of variance. When models indicated significant effects, we used Tukey–Kramer post hoc tests to determine which means differed from one another.

## 3. Results

Of the 228 toads that were exposed to 50 infective *R. pseudosphaerocephala* larvae for 12 h, 101 (44.3%) became infected and 127 remained uninfected. Among the 101 infected toads, the number of adult worms found in the lungs ranged from 1 to 26 (median = 5 worms). For comparison, in a sample of 2497 wild cane toads in Australia, *R. pseudosphaerocephala* prevalence was 44.9% and infection intensity ranged from 1 to 282 worms (media = 8 worms) [39]. We examine the factors affecting these infection dynamics elsewhere [22].

### 3.1. Comparisons between infected and uninfected toads

#### 3.1.1. White blood cell concentrations

#### Basophil concentration

The concentration of basophils was higher in larger toads than in smaller conspecifics (*F*_1,252_ = 5.63, *p* = 0.0184, table 1) but was not significantly related to any other variable in the model (all *p* > 0.07, table 1).

#### Eosinophil concentration

**Figure 3.**
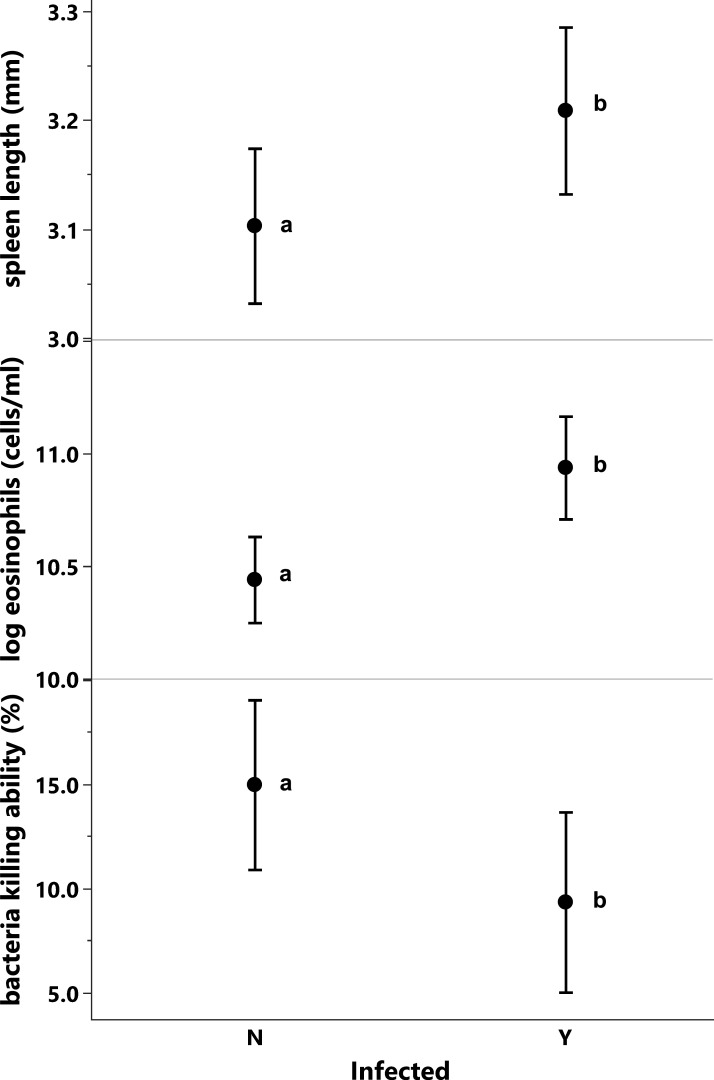
Comparisons of (top) spleen length (corrected for body size), (middle) eosinophil concentration and (bottom) BKA between toads with and without *R. pseudosphaerocephala* lungworm infections. Values are least square means with standard error bars. Within each panel, means with the same letter are not significantly different.

**Figure 4.**
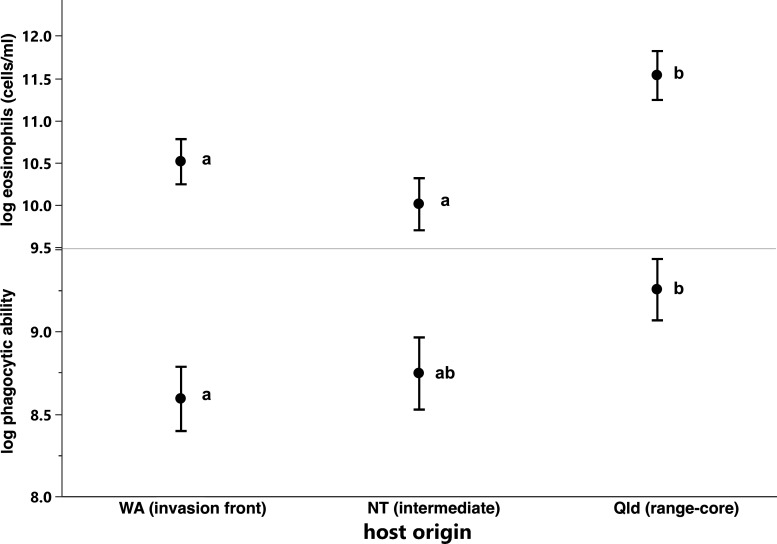
Comparisons of (top) eosinophil concentration and (bottom) phagocytic ability among toads whose parents originated from populations with different invasion histories. Values are least square means with standard error bars. Within each panel, means with the same letter are not significantly different.

The concentration of eosinophils was higher in infected toads than in uninfected ones (2.2 × 10^5^ versus 1.3 × 10^5^ cells ml^−1^; *p* = 0.017, table 1, figure 3) and was higher in toads whose parents had been collected in Qld than in toads whose parents had been collected in the NT or WA (*p* = 0.0006, table 1, figure 4).

#### *L*y*mphocyte conc*entration

The concentration of lymphocytes was not significantly affected by any variables in the model (all *p* > 0.21, table 1).

#### *Neutrophil concentratio*n

The concentration of neutrophils was not significantly affected by any variables in the model (all *p* > 0.29, table 1).

#### 3.1.2. Phagocytosis assay

Phagocytic ability differed among toads from different states (*p* = 0.0331, table 2). Toads whose parents had been collected in Qld had higher phagocytic ability than did toads whose parents had been collected in WA (figure 4). Toads whose parents had been collected in NT had intermediate levels (figure 4). Phagocytic ability was not significantly related to other variables in the model (all *p* > 0.09, table 2).

**Table 2.** Mixed model analyses of the effects of *R. pseudosphaerocephala* infection status and host origin on phagocytic ability, BKA and spleen size. Italic font indicates a significance level of *p* < 0.05.

		phagocytic ability		BKA		spleen length	
effect	d.f.	*F*_234_	*p*	*F*_212_	*p*	*F*_257_	*p*
SVL	1	2.84	0.0932	1.68	0.1967	55.93	***<*** *0.0001*
infected	1	2.04	0.1549	4.16	*0.0427*	5.04	*0.0256*
host origin	2	3.46	*0.0331*	0.62	0.5416	2.41	0.0920
infected × host origin	2	0.66	0.5176	0.37	0.6910	0.29	0.7516

#### 3.1.3. Bacteria-killing assay

The plasma of uninfected toads was better able to kill *E. coli* than was the plasma of infected ones (8.6% versus 14.8%; *p* = 0.0427; table 2, figure 3). BKA was not significantly related to any other variables in the model (all *p* > 0.19, table 2).

#### 3.1.4. Spleen size

After correcting for body size, toads infected with *R. pseudosphaerocephala* had larger spleens than did uninfected conspecifics (3.22 versus 3.07 mm; *p* = 0.0256, table 2, figure 3). Larger toads also had larger spleens (*p* < 0.0001, table 2), but spleen size was not significantly related to toad origin or its interaction with infection status (both *p* > 0.09, table 2).

### 3.2. Infected toads only

#### 3.2.1. White blood cell concentrations

#### Basophils

In infected toads, basophil concentration was not significantly affected by body size, nor by *R. pseudosphaerocephala* burden, worm origin, toad origin nor by any interactions among those variables (all *p* > 0.11, table 3).

**Table 3.** Mixed model analyses of the effects of *R. pseudosphaerocephala* infection intensity, host origin and parasite origin on circulating WBC concentrations in infected toads. Italic font indicates a significance level of *p* < 0.05.

		basophils		eosinophils		lymphocytes		neutrophils	
effect	d.f.	*F*_37_	*p*	*F*_37_	*p*	*F*_37_	*p*	*F*_37_	*p*
SVL	1, 37	2.08	0.1575	0.30	0.5853	0.83	0.3687	0.72	0.4032
no. *Rhabdias*	1, 37	1.07	0.3068	2.99	0.0922	0.25	0.6219	0.01	0.9265
host origin	2, 37	1.46	0.2450	4.89	*0.0130*	0.19	0.8300	0.73	0.4908
parasite origin	2, 37	1.51	0.2335	0.06	0.9420	0.33	0.7197	0.63	0.5377
no. *Rhabdias* × host origin	2, 37	2.30	0.1140	2.05	0.1434	2.83	0.0719	3.33	*0.0468*
no. *Rhabdias* × parasite origin	2, 37	2.78	0.0750	0.25	0.7801	0.02	0.9780	0.13	0.8764
host origin × parasite origin	4, 37	0.45	0.7727	0.79	0.5417	2.00	0.1144	0.68	0.6128
no. *Rhabdias* × host origin × parasite origin	4, 37	2.03	0.1104	1.54	0.2102	3.28	*0.0214*	1.65	0.1814

#### Eosinophils

Eosinophil concentrations differed among toads from different states (*F*_2,37_ = 4.89, *p* = 0.013, table 3). Toads whose parents were collected in Qld had the highest eosinophil levels, NT toads had the lowest and WA toads had intermediate levels. Eosinophil concentration was not significantly affected by other variables or interactions in the models (all *p* > 0.09, table 3).

#### Lymphocytes

**Figure 5.**
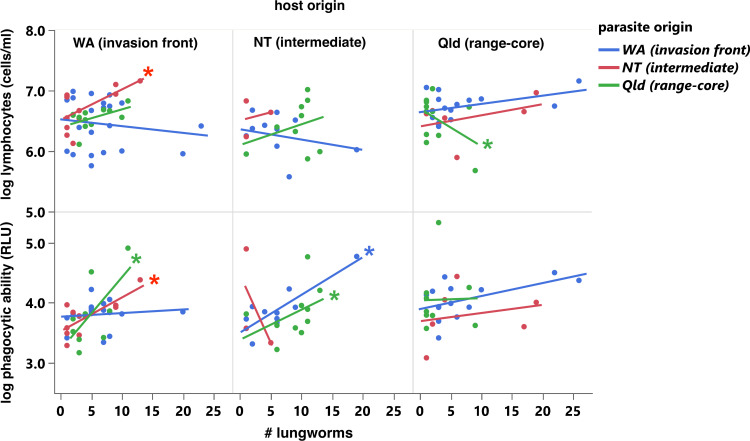
Effects of *Rhabdias pseudosphaerocephala* infection intensity, host origin and parasite origin on (top) lymphocyte concentration; (bottom) phagocytosis ability of cane toads experimentally infected with *R. pseudosphaerocephala* lungworms. Asterisks indicate prediction lines with non-zero slope.

Lymphocyte concentrations in infected toads were affected by the three-way interaction between *R. pseudosphaerocephala* burden, toad origin and parasite origin (*F*_4,37_ = 3.28, *p* = 0.0214, table 3, figure 5). In toads whose parents had been collected in WA, lymphocyte concentrations increased with infection intensity when the animals were infected with allopatric NT parasites. In toads whose parents had been collected in Qld, infection with their sympatric Qld parasites resulted in an increase in lymphocytes as parasite burden increased. When these NT-sourced toads were infected with WA or NT parasites, lymphocytes declined with parasite burden.

#### Neutrophils

**Figure 6.**
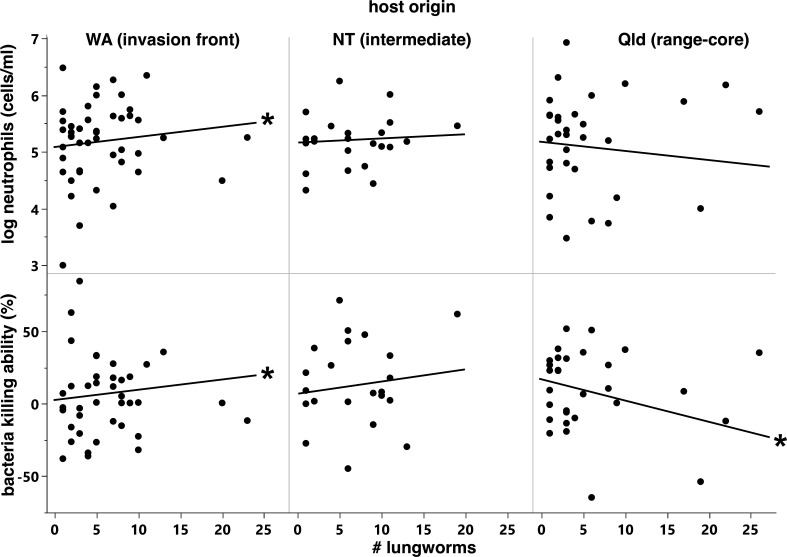
Relationships between *R. pseudosphaerocephala* infection intensity and (top) neutrophil concentration and (bottom) BKA in cane toads whose parents were collected from (left) Western Australia, (centre) Northern Territory (NT) and (right) Queensland (Qld). Asterisks indicate prediction lines with non-zero slopes.

The concentration of neutrophils in infected toads was affected by a significant two-way interaction between infection intensity and toad origin (*F*_2,37_ = 3.33, *p* = 0.0468, table 3, figure 6). In toads whose parents had been collected in WA, neutrophil concentration increased with the level of *R. pseudosphaerocephala* infection, but among toads whose parents had been collected in Qld or the NT, neutrophil concentration was unaffected by infection intensity (figure 6).

#### 3.2.2. Phagocytic ability

Phagocytic ability was affected by the three-way interaction between parasite origin, host origin and infection intensity (*F*_4,50_ = 2.90, *p* = 0.0393) and post hoc examination revealed complex sets of relationships (figure 5). In toads whose parents had been collected from WA and NT, phagocytic ability increased with parasite burden when infected with allopatric lungworms but not when infected with sympatric lungworms (figure 5). In toads whose parents had been collected from Qld, phagocytic ability was independent of infection intensity, regardless of parasite origin (figure 5).

#### 3.2.3. Bacteria-killing ability

The ability of plasma from infected toads to kill *E. coli* was affected by a significant two-way interaction between infection intensity and toad origin (*F*_2,31_ = 5.56, *p* = 0.0086, table 4, figure 6). BKA increased with the level of *R. pseudosphaerocephala* infection in toads whose parents had been collected in WA but decreased in toads whose parents had been collected in Qld. In the offspring of toads from NT, BKA was independent of infection intensity.

**Table 4.** Mixed model analyses of the effects of *R. pseudosphaerocephala* infection intensity, host origin and parasite origin on phagocytic ability, BKA and spleen size. Italic font indicates a significance level of *p* < 0.05.

		phagocytic ability		BKA		spleen length	
effect	d.f.	*F*_29_	*p*	*F*_31_	*p*	*F*_38_	*p*
SVL	1, 50	3.05	0.0914	0.03	0.8618	26.73	*<0.0001*
no. *Rhabdias*	1, 50	0.55	0.4647	2.18	0.1498	7.70	*0.0085*
host origin	2, 50	1.81	0.1810	2.58	0.0917	0.74	0.4861
parasite origin	2, 50	1.40	0.2635	0.46	0.6326	2.13	0.1331
no. *Rhabdias* × host origin	2, 50	2.57	0.0938	5.56	*0.0086*	0.63	0.5397
no. *Rhabdias* × parasite origin	2, 50	2.89	0.0716	0.35	0.7051	1.28	0.2900
host origin × parasite origin	4, 50	2.59	0.0575	1.00	0.4232	1.60	0.1932
no. *Rhabdias* × host origin × parasite origin	4, 50	2.90	*0.0393*	1.58	0.2045	1.87	0.1356

#### 3.2.4. Spleen size

**Figure 7.**
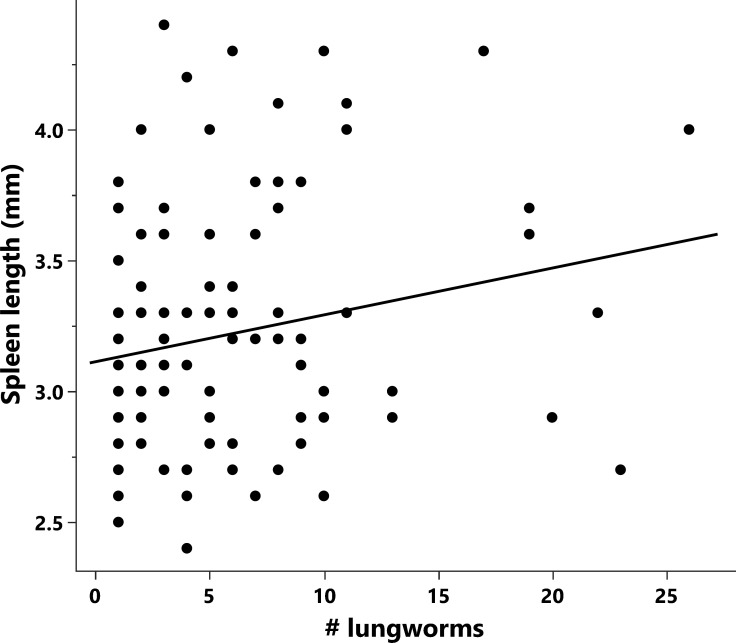
Effects of *R. pseudosphaerocephala* infection intensity on the spleen size of infected cane toads.

Spleen size increased with toad body size (*F*_1,38_ = 26.73, *p* < 0.0001; table 4) and with infection intensity (*F*_1,38_ = 7.70, *p* = 0.0085; table 4, figure 7), regardless of the geographic origin of toads or lungworms.

## 4. Discussion

The immune system of *Rhinella* species is becoming increasingly well studied. The roles played by stress, glucocorticoids and other hormones in how and when immune responses are upregulated, the cytokine cascades involved and the genes ultimately expressed are becoming elucidated [27,40–42]. The role of invasion in changing stress-sensitivity in cane toads, resulting in shifts in their immune regulation is also becoming evident [18,28,43]. Toads at the invasion front are often less responsive to stressors and some immune responses may be dampened as a result [17,28,43]. However, trade-offs among immune components are also apparent and may result in other responses becoming elevated at the invasion front [6,20,25].

Studies comparing immune responses typically use standardized challenges, such as lipopolysaccharide, to activate the immune system. Lipopolysaccharide signals the presence of bacteria without introducing the pathological effects of an infection and the response it elicits in cane toads often differs between invasive and established populations [17,18,25]. Assessing immune reactions using a living parasite that has evolved to evade or modify a particular hosts immune system can add realism to a study [25] but also introduces complexity to the interpretation. Our experimental cross-infection experiment identified shifts in immune measures among cane toads after three to five month lungworm infections, as well as patterns in the ways in which host−parasite interactions have been affected by invasion. Although some of our findings are straightforward and intuitive, others are less amenable to simple interpretation.

### 4.1. Immune system reactions to infection

Our first aim in this study was to determine which immune measures of cane toads responded to infection with lungworms. Differences in spleen sizes suggest that infection results in a general upregulation of immune function. The spleen is a major centre of immune activity in amphibians. It produces WBCs and is a main site for antigen presentation [44]. During immune challenge or parasite infection in toads, spleens enlarge [45] and cytokine production and gene expression activity in the spleen increases [41]. Because adult *R. pseudosphaerocephala* are several millimetres long and their mouthparts penetrate blood vessels in the lungs, they can provoke immune responses either through antigen presentation or by damaging tissue. Thus, the presence of *R. pseudosphaerocephala*, especially in large numbers, stimulates an increase in spleen activity and thus in size.

The magnitude of increase in spleen size provides an index of increased activities such as synthesis of cellular and humoral immune products [27,28,41,45]. Eosinophils appear to be important, based on their higher circulating levels in infected toads. Eosinophils play multiple roles in host defence against helminth parasites, either via direct cytotoxic effects through release of their granules or through modulation of other immune pathways [46,47]. Nonetheless, although infected toads had more eosinophils than did uninfected toads, eosinophil numbers did not increase further with parasite intensity. That is, even a few *R. pseudosphaerocephala* elicited an increase in eosinophils. Metazoan parasites such as *R. pseudosphaerocephala* typically provoke a different suite of immune responses than do bacterial or viral pathogens [48]. Parasite infections typically result in increased levels of eosinophils and basophils, whereas bacterial and viral infections are associated with increased levels of neutrophils and lymphocytes [49].

BKA was lower in toads infected with *R. pseudosphaerocephala* than in uninfected ones. To establish and maintain infections, parasites have abilities to evade detection by or withstand attack from host immune defences [50]. Parasite modulation of host immune responses can be complex and may involve mechanisms such as interference with cytokine pathways or production of microRNAs that can manipulate host gene function [51–54]. It is unclear if the reduced BKA we observed in infected toads is a result of direct modulation by *R. pseudosphaerocephala* or if it is the result of resource trade-offs with other unmeasured immune components (see below).

### 4.2. Effects of host and parasite invasion histories

The second aim of our study was to determine whether the geographic origin of the host or parasite (i.e. from the range core to the invasion front within the toads’ invaded range), affected immune responses. Our fundamental predictions were that (i) toads whose parents were collected from the invasion front would produce weaker immune responses; and (ii) parasites from the invasion front would provoke weaker immune responses. The analyses comparing infected and uninfected toads revealed significant main effects of toad origin on eosinophil levels and on phagocytic ability. In both cases, these immune measures were lower in invasion front (WA) toads than in toads from the longest-established populations in the range core (Qld). Note that these were not different responses to the presence of *R. pseudosphaerocephala* (which would have been indicated by significant infection × toad origin interactions), but rather were differences in the standing, constitutive immune configurations of toads whose parents had been collected at different sites along the toad invasion trajectory. Notably, in both cases where these differences arose, toads whose parents were collected from the invasion front had lower levels than toads whose parents were collected from the range core.

In infected toads, the rates at which neutrophil concentration and BKA changed with infection level differed among toads whose parents had been collected from different areas. Toads whose parents came from populations where individuals are relatively sedentary (i.e. Qld [55]) exhibited reduced levels of both immune measures as the number of *R. pseudosphaerocephala* in their lungs increased. In contrast, toads whose parents were collected from populations closer to the invasion front (WA and NT) tended to increase levels of neutrophils and BKA as infection levels rose. This contrast suggests that at least in regard to these two measures, invasion-front toads commit more resources to immune products when fighting *R. pseudosphaerocephala* infection, not less. However, these responses may still represent economical alternatives to other reactions that are even more costly [3,7,20].

The geographic origin of parasites had less influence than did host origin on the immune measures we made. Parasite origin had a large impact on the success of our experimental infections—invasion-front parasites were most infective, regardless of host origin [22]. On this basis we predicted that parasites whose ancestors were collected from invasion-front populations of cane toads would provoke weaker immune responses than did parasites whose ancestors were collected from range-core populations of toads. Instead, we found that although parasite origin did influence two measures, it was in conjunction with infection intensity and toad origin. Thus, rather than a wholesale shift in parasite virulence with invasion history, local adaptation may underlie these geographic effects. The immune reaction elicited by adult *R. pseudosphaerocephala* in the lungs may depend upon the frequency with which the two genotypes (of host and parasite) encounter each other [56].

The immune responses that we observed may not all be functionally linked to the elimination of adult *R. pseudosphaerocephala* in the lungs. Instead, some of those responses may arise through trade-offs or re-allocations with other unmeasured immune components that may be more directly anthelminthic. In keeping with that hypothesis, some immune responses were lower in infected toads than uninfected ones; or decreased as the intensity of *R. pseudosphaerocephala* infection increased. Reduced BKA in infected toads might result from bactericidal resources being reallocated towards products with more anthelminthic properties, such as eosinophils. Furthermore, we measured immune parameters several months after the toads’ first (and only) exposure to infective larvae. Thus, these measures represent a response to an infection established for three to five months. There may be stronger selection on immune defences during the preliminary stages of infection when larvae are penetrating the skin and migrating through host tissue to reach the lungs [21]. Stopping an infection before it can become established may be a better immune strategy than responding to one after the fact. This raises the possibility that some of the immune responses that we observed four months post infection could represent ‘priming’; that is, aimed at preventing re-infection rather than eliminating the existing one [47,57–59]. Microscopic larvae tunnelling through tissues might be more vulnerable to immune products than are large adult *R. pseudosphaerocephala* attached to the lung lining. Eosinophils, for instance, are typically sequestered in tissues and contain granules of pre-formed cytotoxic chemicals. An increase in their production and deployment after an initial *R. pseudosphaerocephala* infection could be an effective means of intercepting and destroying migrating larvae during subsequent encounters.

## 5. Conclusions

The spatial disequilibrium and altered selective landscape arising from invasive spread can provide opportunities to identify which host and parasite traits diverge when longstanding coexistence is disrupted [12,15,21,22]. The several potential roles parasites can play in biological invasions are well recognized and increasingly well studied [16,23,60–65]. Because of the rather unlikely circumstance of the cane toad bringing and maintaining a single native-range parasite to its expanding introduced range across Australia, it is difficult to frame our results into this wider context. Spatial variation in cane toad immune responses have been previously documented. In their introduced ranges in Florida as well as in Australia, cane toads from invasive range-edge populations show different responses to standardized immune challenges (e.g. lipopolysaccharide) than do their conspecifics from long-established range-core populations [17,18]. Differential immune responses to allopatric versus sympatric parasites are also documented in a variety of taxa, but rarely in contexts where host and parasite are invasive species [16,21]. Although factors such as limited sample size and modest familial and spatial sampling may have had influence on our findings, our results demonstrate evidence of both invasion history and a three to five month *R. pseudosphaerocephala* infection on the immune responses of cane toads.

Although toads from the invasion front have dampened overall levels of some immune measures (eosinophil concentration, phagocytic ability), they have some heightened responses to *R. pseudosphaerocephala* infection intensity (neutrophil concentration, BKA)—sometimes dependent on the origin of the parasite (lymphocyte concentration, phagocytic ability). A deeper understanding of the subtleties and complexities of shifts in toad*–R. pseudosphaerocephala* interactions over the course of invasion could be acquired by documenting levels of gene expression in both host and parasite. It would also be useful to investigate molecular changes during the initial stages of infection when *R. pseudosphaerocephala* larvae are burrowing through tissue en route to the lungs. Selection may act most strongly during preliminary contact as the host attempts to prevent an infection from becoming established while the parasite attempts to complete its life cycle.

## Data Availability

Data is available from the Dryad depository at doi:10.5061/dryad.ghx3ffc09 [66]. Supplementary material is available online [67].
